# Development and internal validation of multivariable prediction models for biochemical failure after MRI-guided focal salvage high-dose-rate brachytherapy for radiorecurrent prostate cancer

**DOI:** 10.1016/j.ctro.2021.06.005

**Published:** 2021-06-29

**Authors:** Thomas Willigenburg, Marieke J. van Son, Sandrine M.G. van de Pol, Wietse S.C. Eppinga, Jan J.W. Lagendijk, Hans C.J. de Boer, Marinus A. Moerland, Jochem R.N. van der Voort van Zyp, Max Peters

**Affiliations:** Department of Radiation Oncology, University Medical Center Utrecht, Utrecht, The Netherlands

**Keywords:** Brachytherapy, Focal salvage high-dose-rate brachytherapy, Prediction model, Prostate cancer, Radiotherapy, Recurrence

## Abstract

**Background and purpose:**

Magnetic resonance-guided focal salvage high-dose-rate brachytherapy (FS-HDR-BT) for radiorecurrent prostate cancer (PCa) shows low toxicity rates. However, biochemical failure (BF) after treatment occurs frequently. We developed two prediction models for BF (Phoenix definition) with the aim of enhancing patient counselling before FS-HDR-BT and during follow-up.

**Materials and methods:**

A prospective cohort of 150 radiorecurrent PCa patients treated with FS-HDR-BT between 2013 and 2020 was used for model development and internal validation. Multivariable Cox Proportional Hazards regression was applied. For model 1, only pre-salvage variables were included as candidate predictors. For model 2, additional (post-)salvage characteristics were tested. After calibration, nomograms and webtools were constructed. Finally, three risk groups were identified.

**Results:**

Sixty-one patients (41%) experienced BF. At baseline (model 1), age, gross tumour volume, pre-salvage PSA, and pre-salvage PSA doubling time (PSADT) were predictive of BF. During follow-up (model 2), age, pre-salvage PSA and PSADT, seminal vesicle involvement, post-salvage time to PSA nadir, and percentage PSA reduction were predictive of BF. The adjusted C-statistics were 0.73 (95% CI: 0.66–0.81) and 0.84 (95% CI: 0.78–0.90), respectively, with acceptable calibration. Estimated 2-year biochemical disease-free survival for the low-, intermediate-, and high-risk groups were 84%, 70%, and 31% (model 1), and 100%, 71%, and 5% (model 2).

**Conclusion:**

Two models are provided for prediction of BF in patients with radiorecurrent PCa treated with FS-HDR-BT. Based on pre- and post-salvage characteristics, we are able to identify patients with a high risk of BF. These findings can aid patient counselling for FS-HDR-BT.

## Highlights

• Biochemical failure rates after focal salvage high-dose-rate brachytherapy are high.
• Evidence on predictors for failure is limited to whole-gland salvage radiotherapy.
• Two prediction models for biochemical failure were developed and internally validated.
• Based on pre- and post-salvage characteristics we can identify high-risk patients.
• These models can be used for patient counselling at baseline and during follow-up.

## Introduction

Advances in prostate cancer (PCa) treatment have increased cure rates. However, still up to 50% of high-risk PCa patients treated with radiotherapy develop a recurrence within 10 years of treatment [1], [2], [3]. These recurrences are often confined to the prostate and frequently located at the site of the primary index lesion [4], [5]. Nowadays, recurrences can be assessed at an earlier stage with prostate specific membrane antigen positron emitting tomography CT (PSMA-PET-CT) [6], [7]. In this setting, focal therapy, targeting the recurrent lesion while sparing healthy prostate tissue, is an attractive treatment option with the aim of postponing initiation of androgen deprivation therapy (ADT) [5], [8]. The main potential advantage of focal over whole-gland salvage treatments is the reduced chance of side-effects and quality of life deterioration, without affecting oncological outcomes [9], [10], [11], [12], [13], [14], [15].

One of the treatment options for radiorecurrent PCa is magnetic resonance imaging (MRI)-guided focal salvage high-dose-rate brachytherapy (FS-HDR-BT) [10], [11]. In previous studies, we found that around 50% of patients treated with single fraction FS-HDR-BT show biochemical failure (BF) within 2.5 years, caused by either local recurrences, regional recurrences, metastatic disease, or a combination [11]. While several studies have been published on predictive factors for BF after whole-gland salvage radiotherapy treatments [16], [17], [18], no studies have been published in patients undergoing focal salvage radiotherapy. Due to differences in patient-, tumour-, and treatment-characteristics, the results from whole-gland salvage studies are not directly applicable to FS-HDR-BT. In the current study we evaluated the predictive value of several pre- and post-salvage variables for BF after FS-HDR-BT for radiorecurrent PCa. Two models were developed, (1) with the aim of enhancing patient selection, based on pre-salvage characteristics, and (2) including additional (post-)salvage characteristics, with the aim of identifying patients at high-risk of BF during follow-up to support patient guidance and counselling.

## Materials and methods

### Patient selection

For this study we prospectively included 150 patients treated with FS-HDR-BT for localized radiorecurrent PCa between July 2013 and January 2020 at the Radiotherapy of the University Medical Center Utrecht (UMCU). Initially, patients were treated within an institutional review board (IRB)-approved feasibility study (Netherlands Trial Register number NTR6123), following the criteria: PSA level ≤ 10 ng/mL, PSA doubling time (PSADT) ≥ 12 months, tumour stage (MRI) ≤ T2c, and acceptable urinary function (International Prostate Symptom Score < 15). Because of favourable toxicity results after 2 years of inclusion, patients beyond the initial inclusion criteria were treated off-protocol. In February 2018, a subsequent phase II study initiated (‘PRostatE Cancer MRI guided focal SalvagE high-dose-rate brachytherapy’, or PRECISE; NTR7014). This study expanded the inclusion criteria from the feasibility study: PSA ≤ 20 ng/mL, PSADT ≥ 9 months, and tumour stage ≤ T3b. All study patients provided written informed consent. A waiver from the IRB was obtained for patients treated off-protocol. Study and treatment details have been described previously [11], [19]. An overview of the three study groups, including inclusion criteria, is presented in Supplementary File A.

### Pre-treatment procedures

Patients underwent pre-treatment 3 T multiparametric (mp) MRI (including T2-weighted (T2W), diffusion-weighted (DWI), and dynamic contrast enhanced (DCE) sequences) and ﻿18F-Choline-PET-CT (n = 14, between 2013 and 2015) or 68 Ga-PSMA-PET-CT (n = 136, from 2015 onwards) scans. Initially, biopsies were performed in all patients (n = 88), either systematically (n = 21) or PET-CT/MRI-targeted (n = 67). However, since the accuracy of Gleason score assessment is debated in irradiated prostate tissue and because biopsies were predominantly positive, biopsies were no longer performed from the end of 2017 onward [20], [21], [22], [23], [24].

A dose of 19 Gray (Gy) was prescribed to the clinical target volume (CTV), which consisted of the MRI- and PET-CT-visible lesion (gross tumour volume [GTV]) plus a 5 mm margin. The GTV was delineated on MRI using the combination of T2W, DWI, and DCE sequences and the PET-CT image. In case the GTV only partially overlapped between the different scans/sequences, the GTV was delineated such that it included the entire suspected area on all sequences. The planning target volume (PTV) was equal to the CTV. Dose constraints to organs at risk were according to protocol and included rectum D1cc and bladder D1cc < 12 Gy, and urethra D10% < 17.7 Gy [11].

### Follow-up and outcome assessment

Follow-up consisted of outpatient clinical visits combined with PSA measurements at 1 and 3 months, every three months the first year, biannually the second year, and annually thereafter up to 10 years. The outcome, BF, was defined according to the Phoenix definition (PSA nadir + 2 ng/mL). In case of BF, follow-up imaging was performed with Ga68-PSMA-PET-CT to assess loco-regional recurrence and/or metastatic disease.

### Candidate variables for model building

To minimize the risk of overfitting, a sample size calculation was performed up front to calculate the number of candidate predictors allowed for multivariable testing. Assuming a 0.05 acceptable difference in apparent and adjusted R-squared, an expected R-squared of 0.15, an overall event rate of 0.2 (200 events per 1000 person-years follow-up), and a shrinkage factor of 0.8, would allow for seven candidate variables with 150 patients and 61 events [25]. For model 1, six candidate variables were selected for multivariable testing based on clinical knowledge and literature [12], [17], [18]. For model 2, three additional variables were tested, thereby accepting a small increase in chance of overfitting. For model 1, the variables assessed pre-salvage included: age at FS-HDR-BT, seminal vesicle involvement, GTV (cm^3^), PSADT (months), PSA (ng/mL), and MRI-based T-stage (T1, T2, and T3 based on NCCN criteria). PSADT was obtained using the Memorial Sloan Kettering Cancer tool (available via: https://www.mskcc.org/nomograms/prostate/psa_doubling_time). For model 2, CTV D95% (dose to 95% of the CTV, in Gy), time to PSA nadir (months) and PSA reduction (ratio between pre-salvage PSA and PSA nadir, in %) were added.

### Statistical analysis

#### Baseline characteristics and survival

Normally distributed determinants are presented as mean (± standard deviation [SD]). Skewed variables are presented as medians with interquartile ranges (IQR). Frequencies and percentages are used for categorical data. The Kaplan-Meier method was used to estimate biochemical disease-free survival (bDFS). For comparisons between groups, the log-rank test was used.

#### Missing data handling

Missing data was considered to be missing at random. Multiple imputation by chained equations was used to impute missing data, creating 20 imputation datasets. All predictors listed above, additional patient and treatment characteristics listed in Supplementary File B, the outcome, and the cumulative baseline hazard, calculated with the Nelson-Aalen function, were included in the imputation procedure [26], [27]. All subsequent modelling steps were pooled over the 20 imputation datasets.

#### Functional form of continuous predictors

Before fitting the multivariable model, non-linear relationships between continuous predictors and the outcome were assessed visually by plotting the predictors against log-hazard using restricted cubic splines with three knots (10^th^, 50^th^, and 90^th^ percentile). In case of visible non-linearity, spline transformations were tested against linear modelling through univariable and multivariable Cox proportional hazards models (likelihood-ratio test). If model fit improved significantly, a spline-transformation was used. For pre-salvage PSA, a natural logarithm-transformation was used based on literature and model fit in our dataset [28].

#### Model development

In case correlations between candidate variables were ≥ 0.75, the clinically most relevant variable was chosen for multivariable testing. MRI-based T-stage showed high correlation with seminal vesicle involvement (correlation coefficient 0.78). Based on clinical judgement, MRI-based T-stage was therefore excluded from multivariable regression analysis. A multivariable Cox proportional hazards regression model was fitted, providing hazard ratios (HR) with 95% confidence intervals (CI). Stepwise backward elimination was performed, using lowest Akaike’s Information Criteria (AIC) for selection [29]. No interactions were assessed due to the limited sample size.

#### Model assumptions

For both models the assumptions of the Cox proportional hazards model were checked. The proportionality assumption was assessed using Log-Log curves and Schoenfeld residuals for categorical and continuous variables, respectively. Linearity of continuous variables was checked with Martingale residuals. Influential outliers were assessed by calculating dfbeta residuals.

#### Model performance and internal validation

The discriminative ability of the model was assessed using Harrell’s C-statistic. Internal validation was performed through bootstrapping with 2000 resamples for each imputation set, in which all modelling steps were repeated. The optimism of each model and shrinkage factors were calculated, and the β-coefficients and C-statistic were adjusted accordingly. The predictive accuracy of the optimism-corrected models was visualized with calibration plots at 12, 24, and 36 months.

#### Nomogram and risk group construction

For both models a nomogram and webtool were constructed using the optimism-corrected coefficients. Finally, for each model separately, three risk groups were identified on the basis of the 25^th^ and 75^th^ percentile of the linear predictor. The Kaplan-Meier method was used to display the biochemical disease-free survival curves for each risk group.

All statistical analyses were performed using R studio (version 3.6.1, R Foundation for Statistical Computing, Vienna, Austria, https://rstudio.com) and the *survival, survminer, rms, pmsampsize, ggplot2, mice, psfmi*, *DynNom,* and *regplot* packages [30]. Reporting was according to the TRIPOD statement [29].

## Results

### Baseline characteristics and Kaplan-Meier survival analysis

Baseline characteristics are displayed in Table 1. Median (IQR) follow-up time was 25.1 months (13.5–38.1) for all patients and 18.1 months (9.2–29.6) for patients who did not experience BF (censored). Sixty-one patients (40.7%) experienced BF after a median (IQR) of 32.9 months (23.5–43.6). Median bDFS was 29.7 months (95% CI: 25.0–38.6) (Fig. S1 in Supplementary File C).

**Table 1:** Baseline patient-, tumour-, and treatment-related characteristics.

Characteristic			Missing (%)
***Primary treatment***			
Primary treatment, n (%)			0
	*EBRT*	80 (53.3)	
	*LDR brachytherapy*	67 (44.7)	
	*HDR brachytherapy*	3 (2)	
EBRT dose (Gy), median (IQR)		76.0 (71.5–77.0)	12.5
LDR dose (Gy), median (IQR)		145.0 (145.0–145.0)	0
HDR dose (Gy), median (IQR)		19.0 (19.0–38.0)	0
PLND at primary treatment, n (%)		30 (20.0)	0
Initial NCCN risk group, n (%)			5.4
	*Low risk*	27 (18.0)	
	*Intermediate risk*	56 (37.3)	
	*High risk*	59 (39.3)	
ADT use (adjuvant/neoadjuvant), n (%)		30 (20.0)	0
ADT duration (mos.), median (IQR) (n = 30)		36.0 (18.0–36.0)	10
PSA nadir post-primary treatment (ng/mL), median (IQR)		0.56 (0.25–1.10)	3.3

***FS-HDR-BT***			
Pre-salvage PSADT (months), median (IQR)		15.7 (11.6–23.6)	0
Interval between primary and salvage treatment (months), median (IQR)		97 (63–128)	0
Age at FS-HDR-BT (years), mean (±SD)		71.5 (±5.0)	0
Pre-salvage PSA (ng/mL), median (IQR)		4.88 (2.80–6.80)	0
Imaging T-stage at FS-HDR-BT, n (%)			0
	*T1-2a*	45 (30.0)	
	*T2b-2c*	40 (26.7)	
	*T3a-3b*	63 (42.0)	
	*T4*	2 (1.3)	
Gleason at FS-HDR-BT, n (%)			45.4
	*3 + 3 = 6*	14 (9.3)	
	*3 + 4 = 7*	27 (18.0)	
	*4 + 3 = 7*	21 (14.0)	
	*Sum score = 8*	6 (4.0)	
	*Sum score = 9/10*	14 (9.3)	
Tumour location, n (%)			0
	*Base*	21 (14.0)	
	*Midgland*	29 (19.3)	
	*Apex*	21 (14.0)	
	*Combination base/midgland/apex*	31 (20.7)	
	*Seminal vesicle*	23 (15.3)	
	*Prostate body and seminal vesicle*	25 (16.7)	
Seminal vesicle involvement at FS-HDR-BT, n (%)		48 (32.0)	0
GTV at FS-HDR-BT (cm^3^), median (IQR)		3.0 (1.7–5.1)	0.7
CTV at FS-HDR-BT (cm^3^), median (IQR)		8.5 (6.0–12.8)	0
Prostate volume at FS-HDR-BT (cm^3^), median (IQR)		31.4 (25.7–39.6)	0
D95% CTV (Gy), median (IQR)		18.8 (17.4–19.7)	0
V200% CTV (%), median (IQR)		26.3 (18.4–27.9)	0
Post-salvage PSA nadir (ng/mL), median (IQR)		0.76 (0.26–1.30)	0
Post-salvage time to PSA nadir (months), median (IQR)		6.1 (3.6–9.6)	0
Percentage PSA reduction (%), median (IQR)		84.2 (68.3–92.9)	0
Biochemical recurrence, n (%)		61 (40.7)	0
Follow-up time (months), median (IQR)		25.1 (13.5–36.1)	0

*Abbreviations: IQR = interquartile range. SD = standard deviation. EBRT = external beam radiotherapy. LDR = low-dose rate. HDR = high-dose rate. PLND = pelvic lymph node dissection. NCCN = national comprehensive cancer network. ADT = androgen deprivation therapy. PSA = prostate specific antigen. FS-HDR-BT = focal salvage high-dose-rate brachytherapy. PSADT = PSA doubling time. GTV = gross tumour volume. D95% = dose to 95% of the volume. V200% = volume receiving 200% or more of the prescribed dose. CTV = clinical target volume*.

### Cox proportional hazards models

Table 2 presents the results from multivariable Cox regressions for model 1 and 2. At baseline (model 1), four variables were identified as significant predictors of BF: age (HR 0.94), pre-salvage PSA (HR 2.19), GTV (HR 1.05), and pre-salvage PSADT (HR 0.87 and 1.18 for PSADT and PSADT’, respectively). For model 2, six predictors were identified: age (HR 0.92), pre-salvage PSADT (HR 0.89 and 1.16), pre-salvage PSA (HR 4.47), seminal vesicle involvement (HR 1.49), post-salvage time to PSA nadir (HR 0.82), and PSA reduction (HR 0.98). Although seminal vesicle involvement was not statistically significant in model 2 (p = 0.14), its exclusion affected AIC notably and therefore it remained in the model. The ranges of the continuous variables in our dataset are displayed in Supplementary File D.

**Table 2:** Multivariable Cox proportional hazards regression analysis for biochemical recurrence for model 1 and model 2.

	Model 1			Model 2		
Candidate predictor	Corrected* β-coefficient	Corrected* HR (95% CI)	p-value	Corrected*^#^* β-coefficient	Corrected*^#^* HR (95% CI)	p-value
Age (years)	−0.065	0.94 (0.90–0.98)	0.003	−0.087	0.92 (0.87–0.96)	0.0005
Pre-salvage PSADT (months)	−0.14	0.87 (0.83–0.92)	<0.0001	−0.12	0.89 (0.83–0.94)	0.0001
Pre-salvage PSADT’ (months)^$^	0.16	1.18 (1.09–1.27)	<0.0001	0.15	1.16 (1.07–1.26)	0.0004
Pre-salvage PSA (ng/mL) (natural logarithm)	0.78	2.19 (1.50–3.18)	0.0001	1.50	4.47 (2.94–6.80)	<0.0001
Seminal vesicle involvement	X	X	X	0.40	1.49 (0.87–2.55)	0.14
GTV (cm^3^)	0.053	1.05 (1.00–1.11)	0.037	X	X	X
D95% CTV (Gy)	*NA*	*NA*	*NA*	X	X	X
Time to PSA nadir post-salvage (months)	*NA*	*NA*	*NA*	−0.20	0.82 (0.76–0.88)	<0.0001
PSA reduction post-salvage (%)	*NA*	*NA*	*NA*	−0.021	0.98 (0.97–0.99)	0.0003

Baseline survival model 1: S_0_(12) = exp(−12.82); S_0_(24) = exp(−65.71); S_0_(36) = exp(−159.00). Baseline survival model 2: S_0_(12) = exp(−214.58); S_0_(24) = exp(−1869.63); S_0_(36) = exp(−5167.25).

*Abbreviations: HR = hazard rate. CI = confidence interval. PSA = prostate specific antigen. FS-HDR-BT = focal salvage high-dose-rate brachytherapy. PSADT = PSA doubling time. GTV = gross tumour volume. D95%=dose to 95% of the volume. CTV = clinical target volume. S*_*0*_*(t) = baseline survival at time point t.*

* Corrected for optimism with shrinkage factor = 0.845. ^#^Corrected for optimism with shrinkage factor = 0.812. ^$^PSADT is modelled using restricted cubic splines (3 knots at 10^th^, 50^th^, and 90^th^ percentile), resulting in one extra parameter, PSADT’, which is depended on PSADT and can be calculated according to the formula for PSADT’ in Supplementary File E. NA = not applicable. X = excluded using backward elimination based on AIC.

### Calibration and internal validation

**Fig. 1:**
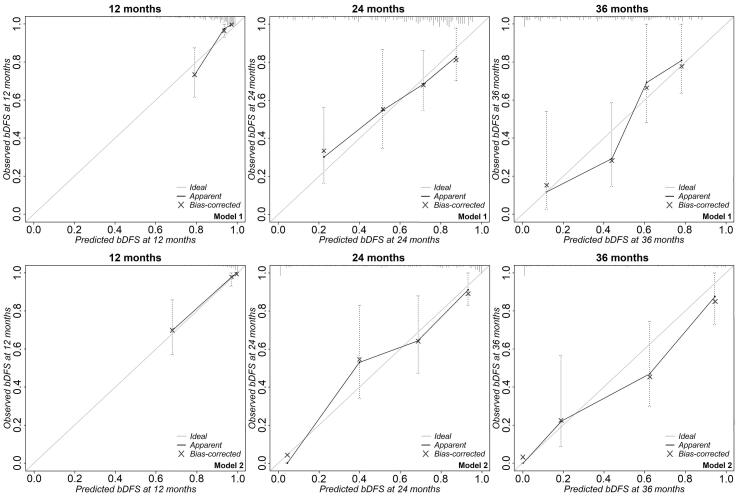
Calibration plots for model 1 (upper row) and model 2 (lower row) depicting the observed (y-axis) versus the predicted probability (x-axis) of biochemical disease-free survival (bDFS) at 12, 24, and 36 months, respectively. Vertical bars indicate the 95% confidence interval. The grey diagonal line depicts the ideal line for complete concordance between observed and predicted probabilities. The blue crosses indicate the optimism-corrected probabilities. (For interpretation of the references to colour in this figure legend, the reader is referred to the web version of this article.)

Calibration curves at 12, 24, and 36 months for both models are depicted in Fig. 1. Calibration was reasonable up to 24 months. Internal validation showed an optimism of 0.15 and 0.19 for model 1 and 2, respectively. The β-coefficients were therefore adjusted with a factor of 0.85 (model 1) and 0.81 (model 2). The C-statistic was adjusted from 0.75 to 0.73 (95% CI: 0.66–0.81) for model 1 and from 0.85 to 0.84 (95% CI: 0.78–0.90) for model 2. The full regression equation for both models can be found in Supplementary file E.

### Nomograms

**Fig. 2:**
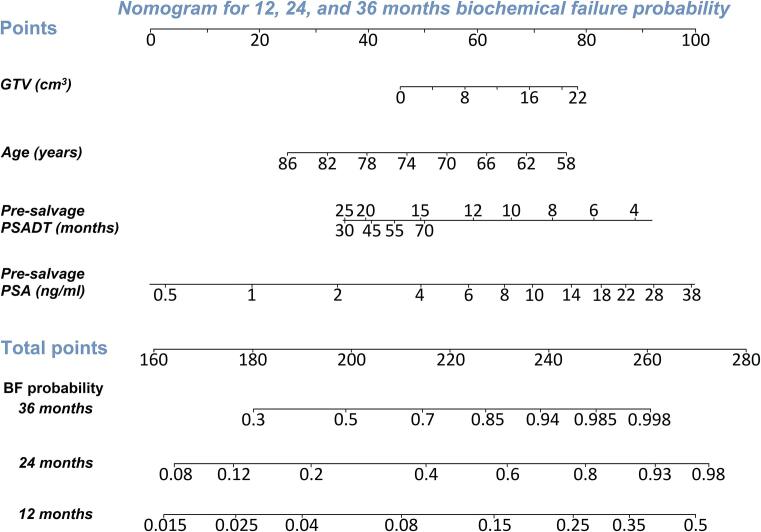
Nomogram based on model 1 for prediction of biochemical failure among patients who underwent FS-HDR-BT. Probabilities of biochemical failure within 12, 24, and 36 months can be calculated. Instruction: Locate the patient’s GTV (cm^3^) of the recurrent prostate cancer lesion on the ‘GTV (cm^3^)’ axis. Draw a line straight upward to the ‘Points’ axis to determine the number of points based on the GTV. Repeat this process for each of the four variables. Sum the points that are received for each of the four predictors (‘Total points’). Finally, draw a line straight down from the ‘Total points’ axis to find the patient’s probability of having biochemical failure within 36, 24, and 12 months, respectively. An interactive version of the nomogram can be used online through: https://fs-hdr-bt-prediction.shinyapps.io/model1/. As an example, a 72-year-old patient with a GTV of 4.0 cm^3^, a PSA-level of 6.0 ng/mL, and a pre-salvage PSADT of 25 months has an estimated 12-, 24-, and 36-months bDFS probability of 95% (95% CI: 93–98%), 78% (95% CI: 70–87%) and 53% (95% CI: 40–71%), respectively. *Abbreviations: GTV = gross tumour volume. PSADT = prostate specific antigen doubling time. PSA = prostate specific antigen. BF = biochemical failure.*

**Fig. 3:**
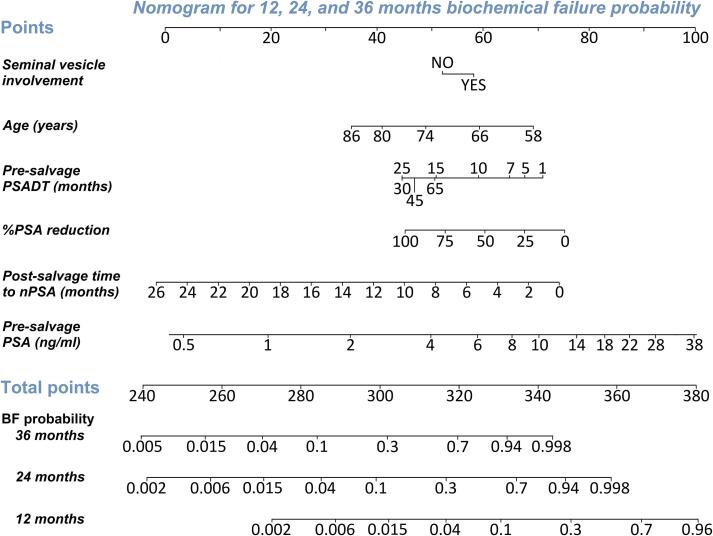
Nomogram based on model 2 for prediction of biochemical failure among patients who underwent FS-HDR-BT. Probabilities of biochemical failure within 12, 24, and 36 months can be calculated. The model can be used online through: https://fs-hdr-bt-prediction.shinyapps.io/model2/. As an example, for the same patient (72 years old, PSA-level 6.0 ng/mL, and a pre-salvage PSADT of 25 months) with no seminal vesicle involvement, PSA nadir after 6 months and a PSA reduction of 90%, the score based on model 2 would be 313, with estimated bDFS probabilities of 98% (95% CI: 96–100%), 80% (95% CI: 71–91%) and 52% (95% CI: 36–74%) at 12, 24, and 36 months. *Abbreviations: PSADT = prostate specific antigen doubling time. PSA = prostate specific antigen. %PSA = percentage PSA. PSA nadir = PSA nadir. BF = biochemical failure.*

**Fig. 4:**
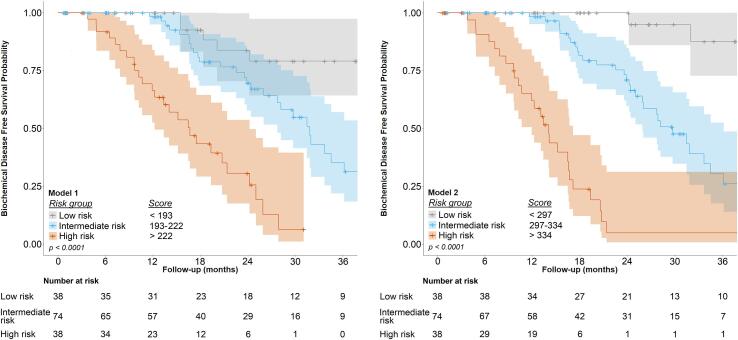
Kaplan-Meier plots for biochemical disease-free survival for low-, intermediate-, and high-risk groups (based on linear predictor/nomogram score), as identified by model 1 (left, nomogram sum scores < 193, 193–222, and > 222, respectively) and model 2 (right, nomogram sum scores < 297, 297–334, and > 334, respectively). Scores are as calculated by the respective nomograms.

The static nomograms for models 1 and 2 are depicted in Fig. 2, Fig. 3, respectively. An exemplary case is included in the figure caption. The Kaplan-Meier curves for bDFS for low-, intermediate-, and high-risk groups, as identified by model 1 (nomogram score < 193, 193–222, and > 222, respectively) and model 2 (nomogram score < 297, 297–334, and > 334, respectively) are shown in Fig. 4. Estimated bDFS at 24 months for low-, intermediate, and high-risk groups was 84%, 70%, and 31% for model 1 (p < 0.0001) and 100%, 71%, and 5% for model 2 (p < 0.0001), respectively. Both models can be used as webtools through: https://fs-hdr-bt-prediction.shinyapps.io/model1/ (model 1) and https://fs-hdr-bt-prediction.shinyapps.io/model2/ (model 2).

## Discussion

This study provides two clinically useful multivariable prediction models for BF in patients with radiorecurrent PCa treated with FS-HDR-BT. Model 1 can be used to support clinical decision making and patient guidance at baseline, while model 2 could be used during follow-up to counsel patients regarding their prognosis and potentially adapt follow-up intensity accordingly.

The predictors in both models and the direction of their effects were mostly as expected. Increased age was associated with a lower hazard of BF. Although causal inference is not applicable in prediction, this could be explained by the potentially longer disease-free survival interval (DFSI) between primary and salvage treatment indicating more indolent tumours. DSFI was longer in elderly patients (median 92 versus 108 months for < 75 years versus ≥ 75 years, respectively). Data on pre-salvage Gleason score is mostly lacking in our cohort, which hinders assessing this relation. Both a higher pre-salvage PSA level and larger GTV were associated with an increased hazard. Both indicate higher tumour load and were therefore expected to be correlated with BF. For pre-salvage PSADT, which was non-linearly related to the outcome, hazard decreased with longer doubling times. This was expected given previous reports [17]. However, from approximately 32 months onward, the hazard increased slightly again, as displayed by a HR of 1.18 for PSADT’. PSADT was ≥ 32 months in only 19 patients (12.7%). Median post-primary PSA nadir, post-salvage PSA nadir, and pre-salvage PSA were higher in these patients compared to those with a PSADT of < 32 months (1.1 vs 0.5 ng/mL, 0.9 vs 0.6 ng/mL, and 6.1 vs 4.6 ng/mL, respectively), but the percentage of patients classified as high-risk (NCCN) at primary treatment was comparable (39% vs 42%). Therefore, we have no clear explanation, and these findings might be caused by the limited sample size. Seminal vesicle involvement, which is a sign of extensive disease, was associated with an increased hazard of BF. A longer post-salvage time to PSA nadir was associated with a lower hazard, potentially reflecting tumour biology (a faster response after radiotherapy could be a sign of more malignant/dedifferentiated PCa) as previously observed [31]. Finally, a larger reduction in PSA level was protective of BF.

Several studies have identified predictors for BF in patients with radiorecurrent PCa treated with focal or whole-gland salvage high-intensity focused ultrasound (HIFU), low-dose rate brachytherapy (LDR-BT), and cryotherapy [17], [32], [33], [34]. However, it is questionable to what extent predictors from whole-gland salvage studies are applicable to focal salvage treatments. Spiess et al. reported a risk stratification model in a whole-gland salvage cryotherapy cohort (n = 132), using the Phoenix definition of BF [33]. Upon multivariable analysis, post-salvage PSA nadir and pre-salvage Gleason score were identified as predictors for BF. PSA nadir was also identified as a predictor of BF after salvage whole-gland HIFU in a small cohort of 50 patients [34]. Peters et al. showed that DFSI between primary and salvage treatment, T-stage before salvage, prostate volume (cm^3^), PSA, and PSADT were predictors of BF in patients treated with focal salvage HIFU [17]. This model shows overlap with our model, indicating that pre-salvage PSA and PSADT are strong predictors for BF after focal salvage treatment for radiorecurrent PCa. While we did not investigate the predictive value of PSA nadir alone, we did incorporate it in our model by using PSA reduction. We argue that this might be a better predictor than PSA nadir, given its dependence on pre-salvage PSA. Furthermore, PSA nadir is also influenced by other factors, such as prostate volume [12]. In another study by Peters et al., univariable analysis showed that age was associated with BF in 62 patients treated with whole-gland brachytherapy. Upon multivariable analysis, age was excluded [18]. This is potentially explained by the limited sample size that was used. It could also be that age and DSFI are associated, as explained in the previous paragraph, and that the effect of age disappears when corrected for DSFI (or vice versa). However, due to the limited sample size we chose not to include DSFI as a candidate predictor. We did not assess pre-salvage Gleason score as a potential predictor, as biopsies were not performed from the end of 2017 onwards (leading to 45.4% missing values). Also, while some have identified variables from the primary tumour and/or treatment as predictors, we did not investigate any primary tumour characteristics because of our limited sample size and missing data in these characteristics. Furthermore, the predictive value of these variables in focal salvage studies seems limited [17]. With an extended sample size and follow-up, we could potentially investigate the added value of some of these predictors.

There are several strengths to our study. Missing data for candidate pre-salvage predictors was very low (0.7%) due to prospective data collection. The inclusion of patients treated off-protocol also makes the study sample more representative and increases external validity. Furthermore, candidate predictors for multivariable analysis were selected based on literature and clinical knowledge rather than by performing univariable analysis, thereby minimizing the occurrence of type-I errors [29]. The online dynamic nomograms we created are helpful tools to quickly assess and visualize individual predicted bDFS.

The study has some limitations. First, external validation of this model is necessary. Several other focal salvage strategies have been described, all with minor differences with respect to eligibility of patients. Therefore, such cohorts offer an opportunity for external validation. Especially since both models use predictors that are known to be related to PCa progression and none of them are treatment specific. External validation of our models could lead to adjustment of these models and thereby improve predictive accuracy and be applicable to other focal salvage modalities. Although evidence is still scarce and mainly limited to the primary treatment setting, fractionated salvage treatment (i.e., 2x13.5 Gy) might improve oncological outcomes in recurrent prostate cancer patients [35], [36], [37]. Despite taking into account the sample size, some overfitting is indicated by the suboptimal shrinkage factors of 0.85 and 0.81, indicating 15% and 19% optimism, respectively. Furthermore, limiting the number of candidate variables might have led to missing important predictors, such as DSFI [17]. Consequently, the C-statistic of 0.73 of the first model might be improved by including other potential predictors when sample size has increased. Third, length of follow-up was relatively short with a median of 25.1 months, thus the models perform optimal within a timeframe of approximately two years. Fourth, tumour volume was based on the delineated GTV. Although GTV delineation was based on mpMRI and PSMA PET-CT, which improves the estimation of tumour volume compared to mpMRI alone [38], interobserver variability due to the lack of delineation guidelines will be present and influences the accuracy and predictive value of this variable.

## Conclusions

This study provides two models for BF prediction in patients with radiorecurrent PCa treated with FS-HDR-BT. Our findings support that both pre- and post-salvage PSA characteristics (PSA level, PSADT, time to PSA nadir, and PSA reduction) are important predictors of BF, in addition to age, tumour volume, and seminal vesicle involvement. These models could aid patient selection, counselling, and guidance at baseline and during follow-up. Potentially, these models can also be used for other salvage techniques, for which external validation remains necessary.

## Funding

This research has been partly funded by the Dutch Cancer Society (The Netherlands, project number 10932). The funding source had no involvement in the design and conduct of the study, the collection, management, analysis, and interpretation of the data, nor in the preparation, review, or approval of the manuscript.

## Declaration of Competing Interest

The authors declare that they have no known competing financial interests or personal relationships that could have appeared to influence the work reported in this paper.

## References

[1] Ward J.F., Pagliaro L.C., Pisters L.L. (2008). Salvage therapy for radiorecurrent prostate cancer. Curr Probl Cancer.

[2] Zelefsky M.J., Kuban D.A., Levy L.B., Potters L., Beyer D.C., Blasko J.C. (2007). Multi-institutional analysis of long-term outcome for stages T1–T2 prostate cancer treated with permanent seed implantation. Int J Radiat Oncol Biol Phys.

[3] Zumsteg Z.S., Spratt D.E., Romesser P.B., Pei X., Zhang Z., Polkinghorn W. (2015). The natural history and predictors of outcome following biochemical relapse in the dose escalation era for prostate cancer patients undergoing definitive external beam radiotherapy. Eur Urol.

[4] Cellini N., Morganti A.G., Mattiucci G.C., Valentini V., Leone M., Luzi S. (2002). Analysis of intraprostatic failures in patients treated with hormonal therapy and radiotherapy : Implications for conformal therapy planning. Int J Radiat Oncol Biol Phys.

[5] Pucar D., Hricak H., Shukla-Dave A., Kuroiwa K., Drobnjak M., Eastham J. (2007). Clinically significant prostate cancer local recurrence after radiation therapy occurs at the site of primary tumor: Magnetic resonance imaging and step-section pathology evidence. Int J Radiat Oncol Biol Phys.

[6] Bluemel C., Krebs M., Polat B., Linke F., Eiber M., Samnick S. (2016). 68Ga-PSMA-PET/CT in patients with biochemical prostate cancer recurrence and negative 18F-Choline-PET/CT. Clin Nucl Med.

[7] Afshar-Oromieh A., Zechmann C.M., Malcher A., Eder M., Eisenhut M., Linhart H.G. (2014). Comparison of PET imaging with a (68)Ga-labelled PSMA ligand and (18)F-choline-based PET/CT for the diagnosis of recurrent prostate cancer. Eur J Nucl Med Mol Imaging.

[8] Zumsteg Z.S., Spratt D.E., Romesser P.B., Pei X., Zhang Z., Kollmeier M. (2015). Anatomic patterns of recurrence following biochemical relapse in the dose-escalation era for prostate patients undergoing external beam radiotherapy. J Urol.

[9] Khoo C.C., Miah S., Connor M.J., Tam J., Winkler M., Ahmed H.U. (2020). A systematic review of salvage focal therapies for localised non-metastatic radiorecurrent prostate cancer. Transl Androl Urol.

[10] Van Son M, Peters M, Moerland M, Kerkmeijer L, Lagendijk J, Van der Voort van Zyp J. Focal salvage treatment of radiorecurrent prostate cancer: A narrative review of current strategies and future perspectives. Cancers (Basel) 2018;10:480. https://doi.org/10.3390/cancers10120480. 10.3390/cancers10120480 PMC6316339 30513915

[11] van Son M.J., Peters M., Moerland M.A., Lagendijk J.J.W., Eppinga W.S.C., Shah T.T. (2020). MRI-guided ultrafocal salvage high-dose-rate brachytherapy for localized radiorecurrent prostate cancer: updated results of 50 Patients. Int J Radiat Oncol.

[12] Stabile A., Orczyk C., Giganti F., Moschini M., Allen C., Punwani S. (2020). The role of percentage of prostate-specific antigen reduction after focal therapy using high-intensity focused ultrasound for primary localised prostate cancer. Results from a Large Multi-institutional Series. Eur Urol.

[13] Tan W.P., ElShafei A., Aminsharifi A., Khalifa A.O., Polascik T.J. (2020). Salvage focal cryotherapy offers similar short-term oncologic control and improved urinary function compared with salvage whole gland cryotherapy for radiation-resistant or recurrent prostate cancer. Clin Genitourin Cancer.

[14] Bomers J.G.R., Overduin C.G., Jenniskens S.F.M., Cornel E.B., van Lin E.N.J.T., Sedelaar J.P.M. (2020). Focal Salvage MR imaging-guided cryoablation for localized prostate cancer recurrence after radiotherapy: 12-Month Follow-up. J Vasc Interv Radiol.

[15] Duijzentkunst D.A.S., Peters M., van der Voort van Zyp J.R.N., Moerland M.A., van Vulpen M. (2016). Focal salvage therapy for local prostate cancer recurrences after primary radiotherapy: a comprehensive review. World J Urol.

[16] Henríquez I., Sancho G., Hervás A., Guix B., Pera J., Gutierrez C. (2014). Salvage brachytherapy in prostate local recurrence after radiation therapy: Predicting factors for control and toxicity. Radiat Oncol.

[17] Peters M., Kanthabalan A., Shah T.T., McCartan N., Moore C.M., Arya M. (2018). Development and internal validation of prediction models for biochemical failure and composite failure after focal salvage high intensity focused ultrasound for local radiorecurrent prostate cancer: Presentation of risk scores for individual patient progn. Urol Oncol Semin Orig Investig.

[18] Peters M., van der Voort van Zyp J.R.N., Moerland M.A., Hoekstra C.J., van de Pol S., Westendorp H. (2016). Development and internal validation of a multivariable prediction model for biochemical failure after whole-gland salvage iodine-125 prostate brachytherapy for recurrent prostate cancer. Brachytherapy.

[19] Maenhout M., Peters M., van Vulpen M., Moerland M.A., Meijer R.P., van den Bosch M.A.A.J. (2017). Focal MRI-guided salvage high-dose-rate brachytherapy in patients with radiorecurrent prostate cancer. Technol Cancer Res Treat.

[20] Grignon D.J., Sakr W.A. (1995). Histologic effects of radiation therapy and total androgen blockade on prostate cancer. Cancer.

[21] Crook J.M., Bahadur Y.A., Robertson S.J., Perry G.A., Esche B.A. (1997). Evaluation of radiation effect, tumor differentiation, and prostate specific antigen staining in sequential prostate biopsies after external beam radiotherapy for patients with prostate carcinoma. Cancer.

[22] Goldstein N.S., Martinez A., Vicini F., Stromberg J. (1998). The histology of radiation therapy effect on prostate adenocarcinoma as assessed by needle biopsy after brachytherapy boost: Correlation with biochemical failure. Am J Clin Pathol.

[23] Prestidge B.R., Hoak D.C., Grimm P.D., Ragde H., Cavanagh W., Blasko J.C. (1997). Posttreatment biopsy results following interstitial brachytherapy in early-stage prostate cancer. Int J Radiat Oncol Biol Phys.

[24] Crook J., Malone S., Perry G., Bahadur Y., Robertson S., Abdolell M. (2000). Postradiotherapy prostate biopsies: what do they really mean? Results for 498 patients. Int J Radiat Oncol Biol Phys.

[25] Riley RD, Ensor J, Snell KIE, Harrell FE, Martin GP, Reitsma JB, et al. Calculating the sample size required for developing a clinical prediction model. BMJ 2020;368:1–12. https://doi.org/10.1136/bmj.m441. 10.1136/bmj.m441 32188600

[26] White I.R., Royston P. (2009). Imputing missing covariate values for the Cox model. Stastics Med.

[27] Moons K.G.M., Donders R.A.R.T., Stijnen T., Harrell F.E. (2006). Using the outcome for imputation of missing predictor values was preferred. J Clin Epidemiol.

[28] Nieboer D., Vergouwe Y., Roobol M.J., Ankerst D.P., Kattan M.W., Vickers A.J. (2015). Nonlinear modeling was applied thoughtfully for risk prediction: The Prostate Biopsy Collaborative Group. J Clin Epidemiol.

[29] Moons K.G.M., Altman D.G., Reitsma J.B., Ioannidis J.P.A., Macaskill P., Steyerberg E.W. (2015). Transparent reporting of a multivariable prediction model for individual prognosis or diagnosis (TRIPOD): Explanation and elaboration. Ann Intern Med.

[30] Team RC. R: A language and environment for statistical computing 2019.

[31] Ray M.E., Thames H.D., Levy L.B., Horwitz E.M., Kupelian P.A., Martinez A.A. (2006). PSA nadir predicts biochemical and distant failures after external beam radiotherapy for prostate cancer: A multi-institutional analysis. Int J Radiat Oncol Biol Phys.

[32] Spiess P.E., Katz A.E., Chin J.L., Bahn D., Cohen J.K., Shinohara K. (2010). A pretreatment nomogram predicting biochemical failure after salvage cryotherapy for locally recurrent prostate cancer. BJU Int.

[33] Spiess P.E., Levy D.A., Mouraviev V., Pisters L.L., Jones J.S. (2013). Predictors of biochemical failure in patients undergoing prostate whole-gland salvage cryotherapy: A novel risk stratification model. BJU Int.

[34] Shah T.T., Peters M., Kanthabalan A., McCartan N., Fatola Y., van der Voort van Zyp J. (2016). PSA nadir as a predictive factor for biochemical disease-free survival and overall survival following whole-gland salvage HIFU following radiotherapy failure. Prostate Cancer Prostatic Dis.

[35] Morton G., McGuffin M., Chung H.T., Tseng C.-L., Helou J., Ravi A. (2020). Prostate high dose-rate brachytherapy as monotherapy for low and intermediate risk prostate cancer: Efficacy results from a randomized phase II clinical trial of one fraction of 19 Gy or two fractions of 13.5 Gy. Radiother Oncol J Eur Soc Ther Radiol Oncol.

[36] Murgic J., Morton G., Loblaw A., D'Alimonte L., Ravi A., Wronski M. (2018). Focal salvage high dose-rate brachytherapy for locally recurrent prostate cancer after primary radiation therapy failure: Results from a prospective clinical trial. Int J Radiat Oncol Biol Phys.

[37] Jiang P., van der Horst C., Kimmig B., Zinsser F., Poppe B., Luetzen U. (2017). Interstitial high-dose-rate brachytherapy as salvage treatment for locally recurrent prostate cancer after definitive radiation therapy: Toxicity and 5-year outcome. Brachytherapy.

[38] Bettermann A.S., Zamboglou C., Kiefer S., Jilg C.A., Spohn S., Kranz-Rudolph J. (2019). [68 Ga- ] PSMA-11 PET / CT and multiparametric MRI for gross tumor volume delineation in a slice by slice analysis with whole mount histopathology as a reference standard – Implications for focal radiotherapy planning in primary prostate cancer. Radiother Oncol.

