# Supplementary material for: Development and internal validation of multivariable prediction models for biochemical failure after MRI-guided focal salvage high-dose-rate brachytherapy for radiorecurrent prostate cancer
Source: Clin Transl Radiat Oncol. 2021 Jun 29;30:7–14. doi: 10.1016/j.ctro.2021.06.005 (PMC8261471; doi:10.1016/j.ctro.2021.06.005)
Supplement: Supplementary data 1 [file mmc1.pdf]

SUPPLEMENTARY FILE A

Study/treatment group	*N*	*Years of inclusion*	*Study specific inclusion criteria*
Feasibility study	30	2013-2017	*Patient characteristics*<br>Age ≥ 18 years;<br>Karnofsky Performance Status ≥ 70;<br>Fit for spinal anaesthesia;<br>International Prostate Symptom Score < 15.<br><br>*Tumour characteristics*<br>Tumour location technically feasible for brachytherapy;<br>Tumour visible MRI and PSMA/choline-PET/CT;<br>Biopsy proven local recurrence;<br>PSA level ≤ 10 ng/mL;<br>PSA doubling time (PSADT) ≥ 12 months;<br>Tumour stage (MRI) ≤ T2c;<br>Recurrence ≥ 2 years after primary radiotherapy;<br>No lymph node and/or distant metastases.
PRECISE study	42	2018-2019	*Patient characteristics*<br>See 'Feasibility study'.<br><br>*Tumour characteristics*<br>Tumour location technically feasible for brachytherapy;<br>Concordance between PSMA-PET/CT and mp-MRI;<br>PSA level ≤2 0 ng/mL;<br>PSA doubling time (PSADT) ≥ 9 months;<br>Tumour stage (MRI) ≤ T3b;<br>Recurrence ≥ 2 years after primary radiotherapy;<br>No lymph node and/or distant metastases.
Non-study	78	2015-2020	*Patient characteristics*<br>See 'Feasibility study'.<br><br>*Tumour characteristics*<br>No specific criteria (i.e., those not eligible for treatment within either the feasibility of the PRECISE study, for example due to higher pre-salvage PSA and/or shorter PSA doubling time).
