# Supplementary material for: Development and internal validation of multivariable prediction models for biochemical failure after MRI-guided focal salvage high-dose-rate brachytherapy for radiorecurrent prostate cancer
Source: Clin Transl Radiat Oncol. 2021 Jun 29;30:7–14. doi: 10.1016/j.ctro.2021.06.005 (PMC8261471; doi:10.1016/j.ctro.2021.06.005)
Supplement: Supplementary data 2 [file mmc2.pdf]

SUPPLEMENTARY FILE B

Additional variables used for multiple imputation via chained equations:

- Primary treatment
    - *EBRT*, *LDR brachytherapy*, or *HDR brachytherapy*
- Pelvic lymph node dissection at primary treatment (yes/no)
- Initial NCCN risk group
    - *Low risk (T1-T2a AND Gleason* ≤ 6 AND PSA < 10 ng/mL), *Intermediate risk (T2b-T2c OR Gleason 7 OR PSA 10-20 ng/mL)*, or *High risk (*≥ T3a OR Gleason ≥ 8 OR PSA > 20 ng/mL)
- ADT use
    - *No, ≤ 36 months*, or *> 36 months*
- PSA nadir post-primary treatment (ng/mL)
- Prostate size on pre-salvage MRI (cm^3)
- Maximum tumour diameter on pre-salvage MRI (mm)
- V200% CTV (%)
- PSA velocity (ng/mL/years)
- Interval between primary and salvage treatment (months)
- Previous salvage treatment (yes/no)
- Time to nadir post-primary treatment (months)
- Deceased (yes/no)
