# Supplementary material for: Development and internal validation of multivariable prediction models for biochemical failure after MRI-guided focal salvage high-dose-rate brachytherapy for radiorecurrent prostate cancer
Source: Clin Transl Radiat Oncol. 2021 Jun 29;30:7–14. doi: 10.1016/j.ctro.2021.06.005 (PMC8261471; doi:10.1016/j.ctro.2021.06.005)
Supplement: Supplementary data 3 [file mmc3.pdf]

SUPPLEMENTARY FILE C

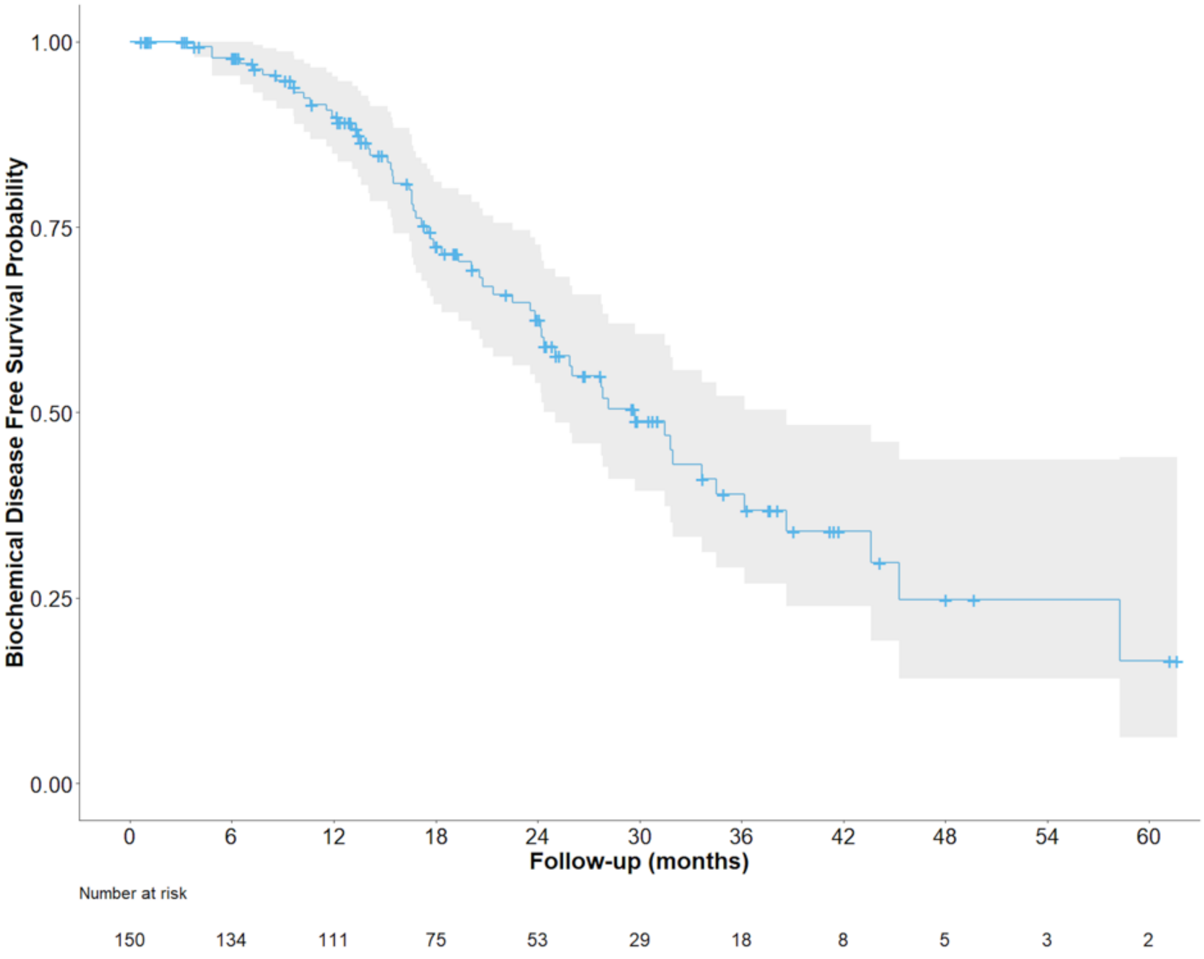

Figure S1 *Kaplan-Meier curve depicting biochemical disease-free survival for the entire FS-HDR-BT group.*
