# Supplementary material for: Development and internal validation of multivariable prediction models for biochemical failure after MRI-guided focal salvage high-dose-rate brachytherapy for radiorecurrent prostate cancer
Source: Clin Transl Radiat Oncol. 2021 Jun 29;30:7–14. doi: 10.1016/j.ctro.2021.06.005 (PMC8261471; doi:10.1016/j.ctro.2021.06.005)
Supplement: Supplementary data 4 [file mmc4.pdf]

SUPPLEMENTARY FILE D

Predictor	***Range in dataset used for model building***
Pre-salvage PSADT (months)	3 – 73 months
Age at FS-HDR-BT (years)	59 – 85 years
Pre-salvage PSA (ng/mL)	0.4 – 39.0 ng/mL
GTV (cm^3)	0.5 – 22 cm^3
Time to PSA nadir post-salvage (months)	1 – 25 months
PSA reduction post-salvage (%)	0 – 100 %

Abbreviations: PSADT = prostate specific antigen doubling time. FS-HDR-BT = focal salvage high-dose-rate-brachytherapy. PSA = prostate specific antigen. GTV = gross tumour volume.
