# Supplementary material for: Development and internal validation of multivariable prediction models for biochemical failure after MRI-guided focal salvage high-dose-rate brachytherapy for radiorecurrent prostate cancer
Source: Clin Transl Radiat Oncol. 2021 Jun 29;30:7–14. doi: 10.1016/j.ctro.2021.06.005 (PMC8261471; doi:10.1016/j.ctro.2021.06.005)
Supplement: Supplementary data 5 [file mmc5.pdf]

SUPPLEMENTARY FILE E

Model 1

The full regression equation for model 1 is as follows:

$$h(t) = h_0(t) * \exp\,(-0.065 * Age \; + \; 0.78 * \ln\,(PSA) \; + \; 0.053 * GTV \; - \; 0.14 * PSADT \; + \; 0.16 * PSADT')$$

Or for calculating the predicted survival probability:

$$s(t) = s_0(t)^{\exp\,(-0.065*Age \, + \, 0.78*\ln\,(PSA) \, + \, 0.053*GTV \, - \, 0.14*PSADT \, + \, 0.16*PSADT')}$$

Where:

$h(t)$ = the expected hazard at time *t*

$h_0(t)$ = the baseline hazard at time *t*

$s(t)$ = the expected survival probability at time t

$s_0(t)$ = the baseline survival at time *t (see Table 2 for baseline survival at 12, 24, and 36 months)*

Age = age in years at time of FS-HDR-BT

ln = natural logarithm

PSA = pre-salvage PSA level in ng/mL

GTV = gross tumour volume in cm^3

PSADT = PSA doubling time in months

PSADT’ can be calculated as follows:

$$PSADT' = \frac{(PSADT - 7.98\,)_+^3 - \; 1.43(PSADT - 15.70)_+^3 + 0.44(PSADT - 33.63)_+^3}{657.9225}$$

Model 2:

$$h(t) = h_0(t) * \exp\,(-0.087 * Age \; + \; 1.50 * \ln\,(PSA) \; + \; 0.40 * Seminal\ vesicle\ involment \; - \; 0.12 * PSADT + \; 0.15 * PSADT' - \; 0.20 * Time\ to\ PSA\ nadir \; - \; 0.021 * \%PSA\ reduction)$$

Or for calculating the predicted survival probability:

$$s(t) = s_0(t)^{\exp\,(-0.087*Age\, +\, 1.50*\ln(PSA)\, +\, 0.40*Seminal\ vesicle\ involvement\, -\, 0.12*PSADT + \, 0.15*PSADT' - \, 0.20*Time\ to\ PSA\ nadir\, -\, 0.021*\%PSA\ reduction)}$$

Where:

h(t) = the expected hazard at time *t*

h_0(t) = the baseline hazard at time *t*

s(t) = the expected survival probability at time t

s_0(t) = the baseline survival at time *t (see Table 2 for baseline survival at 12, 24, and 36 months)*

Age = age in years at time of FS-HDR-BT

ln = natural logarithm

PSA = pre-salvage PSA level in ng/mL

Seminal vesicle involvement = 0 when not applicable, 1 when applicable

Time to PSA nadir = post-salvage time to nadir in months

%PSA reduction = PSA reduction (ratio between pre-salvage PSA and post-salvage PSA nadir) in %

PSADT = PSA doubling time in months

PSADT' can be calculated as follows:

$$PSADT' = \frac{(PSADT - 7.98\,)_+^3 - \; 1.43(PSADT - 15.70)_+^3 + 0.44(PSADT - 33.63)_+^3}{657.9225}$$
